# Solitary Fibrous Tumor of the Retroperitoneal Space

**DOI:** 10.1155/2021/8857274

**Published:** 2021-01-09

**Authors:** Mateusz Marcinek, Konrad Majcherczyk, Marcel Nowak, Aleksander Targoński, Michał Tkocz

**Affiliations:** Department of Urology, Faculty of Medical Sciences in Katowice, Medical University of Silesia, Katowice, Poland, Plac Medyków 1, 41-200 Sosnowiec, Poland

## Abstract

A solitary fibrous tumor develops from mesenchymal cells as a pleural neoplasm, but it is also occasionally reported in extrapleural sites. Retroperitoneal tumors are a group of neoplasms located between muscles and the fascia of the posterior abdominal wall and the parietal peritoneum. Their cytology differs from that of urinary tract organs or adrenals. This case report presents a rare solitary fibrous tumor incidentally found during an ultrasound examination. A 54-year-old male underwent urgent surgery for a tumor located in the left retroperitoneal space. The histologic examination confirmed a solitary fibrous tumor with a 5% Ki67 proliferation index, a 1 MF/10 HPF mitotic activity, and CD34-positive immunostains. A solitary fibrous tumor is a rare retroperitoneal tumor. Its symptoms and signs might resemble those of the classical triad of renal cell carcinoma, although the tumor's growth phase is typically asymptomatic. Intraoperative diagnosis of a solitary fibrous tumor strongly recommends radical excision.

## 1. Introduction

A solitary fibrous tumor (SFT) is a rare type of soft tissue sarcoma that develops from mesenchymal cells [1]. It was described for the first time in 1931 as a pleural neoplasm [2], which is its most common location. Since then, it has also been reported, but very rarely, in several extrapleural sites [1, 3, 4]. Symptoms depend on lesion location. The diagnosis consists of ultrasound, followed by computer tomography (CT) or magnetic resonance imaging (MRI). The standard treatment consists of surgical resection with free resection margins; however, adjuvant chemotherapy might prove beneficial [5]. The final diagnosis is made based on the histopathology report.

This case report presents a rare solitary fibrous tumor incidentally diagnosed on an ultrasound examination.

## 2. Case Presentation

**Figure 1:**
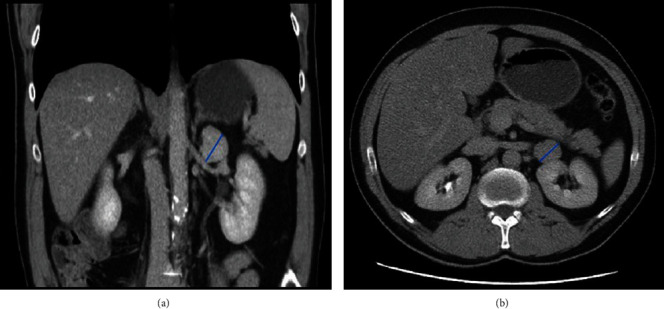
Contrast-enhanced CT revealing the tumor in the left retroperitoneal space: (a) frontal section and (b) cross-section. A blue arrow indicates the diameter of the tumor.

A 54-year-old male, suspected of a left kidney tumor, was urgently referred to our urology clinic. The ultrasound examination incidentally revealed the lesion and reported it as a 3 cm mass in the upper pole of the left kidney. The patient was asymptomatic: he did not report any weight loss, and the lesion was undetectable by palpation. He presented with several associated diseases, including intervertebral disc degeneration, but had no history of surgical treatment. Multiphase contrast-enhanced computer tomography (Figure 1) revealed a 31 × 28 × 31 mm mass at the upper pole of the left kidney, showing strong and relatively uniform contrast enhancement. The lesion was located between the left adrenal and anterior kidney surface. The length of the tumor-kidney contact area was 22 mm. No other abnormalities were detected. Both kidneys were normal and they excreted urine into nondilated pelvicalyceal systems. The chest radiographs were also normal.

The patient was admitted to our Urology Department for urgent surgical treatment of a left kidney tumor. Laboratory tests showed no abnormalities; creatinine level was 1.0 mg/dl (GFR: 82.90 ml/min/1.73 m^2^). At the preanesthetic assessment, the patient was classified as ASA1. Surgery was started using lumbar access. A 3.5 cm tumor was seen at the upper pole of the left kidney without apparent involvement of the kidney or other organs. Gross total resection was performed. The postoperative course was uneventful. The patient was discharged home in good condition three days after the surgery.

**Figure 2:**
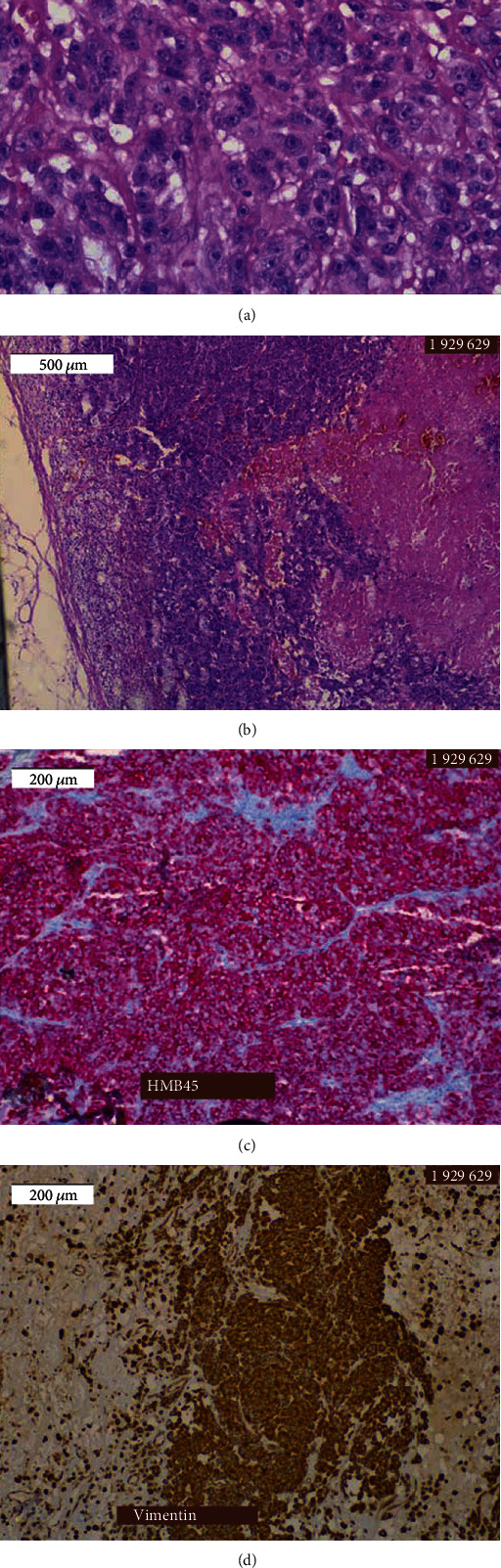
Histological sections of excised tumor. Light microscope examination: (a) hematoxylin-eosin staining, 100x magnification; (b) hematoxylin-eosin staining, 10x magnification; (c) HMB45 staining, 40x magnification; (d) vimentin staining, 40x magnification.

The histologic examination revealed an extrapleural solitary fibrous tumor (35 × 30 × 30 mm) with a Ki67 proliferation index of 5%, a mitotic activity of 1 MF/10 HPF, and positive immunostaining for CD34. There was no histologic evidence of necrosis (Figure 2).

A follow-up examination performed three months after the surgery consisted of laboratory tests, CT of the abdomen, and chest X-ray. No abnormalities were detected. The patient continues to come in for scheduled appointments at our urology clinic.

## 3. Discussion

Retroperitoneal tumors are located between the posterior abdominal muscles, associated fascia, and the parietal peritoneum. They are defined as neoplasms derived from tissues that are not typical of the urinary tract organs or adrenals. The tumors represent approximately 2-3% of all neoplasms [6, 7]. The solitary fibrous tumor (SFT) was described for the first time in 1931 by Klemperer and Rabin as a pleural neoplasm [2]. SFT is a type of soft tissue sarcoma that accounts for about 5% of retroperitoneal sarcomas [8, 9]. Originally, SFT was believed to develop from the mesothelial pleural layer, but electron microscopy and immunohistochemistry revealed that the tumor develops from myofibroblasts [6, 10]. SFT most frequently develops in the pleura, but extrapleural lesions, including mediastinal, cervical, pelvic, and orbital involvements, have also been described [1, 3, 4]. SFTs tend to be well-circumscribed benign lesions originating from multipotent mesenchymal cells [11, 12]. Their malignant recurrences occur quite rarely [12, 13]. Benign forms consist of spindle cells with low mitotic activity and low-to-moderate nuclear atypia. Contrary to this, malignant forms exhibit high cellularity, increased nuclear pleomorphism, and high mitotic activity. The mitotic rate differentiates benign (4 mitoses per 10 high-power fields (HPF)) from malignant tumors [6].

Retroperitoneal tumors might be asymptomatic. If symptoms occur, they tend to be noncharacteristic, depend on tumor size and location, and mainly result from displacement of the neighboring organs. Retroperitoneal lesions grow slowly and thus are detected at late stages [14]. They are hardly palpable. The asymmetric increases in abdominal circumference are frequently the only reason for patients to seek medical advice. Computed tomography or magnetic resonance should always be performed to confirm the diagnosis.

The treatment of choice for retroperitoneal SFTs is radical excision. Since lymph node metastases are infrequent, there is no need for retroperitoneal lymphadenectomy to be performed simultaneously with surgery for the tumor [5]. Distant metastatic spread is rarely observed, but treatment failure includes local recurrence. Nonradical surgery does not improve survival [9]. There are no results of prospective studies to explicitly confirm that radiotherapy reduces the risk of local relapse. It should also be noted that critical organs of the retroperitoneal space make it difficult or impossible to deliver optimum doses of radiation therapy. The role of chemotherapy remains controversial [5].

The final diagnosis is based on histology reports, as specific immunohistochemical staining of surgical specimens plays a critical role in the diagnostic process [15]. Regular postsurgery follow-ups must be scheduled to monitor the patient for local recurrence, using ultrasound and CT, or metastatic spread, using chest X-ray.

## 4. Conclusions

A solitary fibrous tumor usually develops as an asymptomatic or oligosymptomatic lesion. It is rarely diagnosed in the retroperitoneal space. It tends to be found incidentally during an ultrasound examination performed for some other reason. Radical excision is the treatment of choice, but a follow-up at a urology outpatient clinic is strongly recommended.

## Data Availability

Data available on request due to privacy restrictions.
